# Babcock versus Scissor Tensioning for Retropubic Mid-Urethral Slings: Comparing Two Intra-Operative Techniques Through 5 Years of Follow-Up

**DOI:** 10.1007/s00192-024-05916-y

**Published:** 2024-10-01

**Authors:** Erin A. Brennand, Julia Chai, Shannon Cummings, Beili Huang, Taylor Hughes, Allison Edwards, Alison Carter Ramirez, for the Calgary Women’s Pelvic Health Research Group

**Affiliations:** 1 Department of Obstetrics & Gynecology, University of Calgary, North Tower, Foothills Medical Center, 1441 – 29 Street NW, Calgary, AB T2N 4J8 Canada; 2 Department of Community Health Sciences, University of Calgary, Calgary, Canada; 3 Department of Obstetrics & Gynecology, University of Alberta, Edmonton, Canada

**Keywords:** Stress urinary incontinence, Suburethral sling, Tension-free vaginal tape

## Abstract

**Introduction and Hypothesis:**

The objective was to determine if mid-urethral sling (MUS) tensioning with a Mayo Scissor as a sub-urethral spacer compared with a Babcock clamp holding a loop of tape under the urethra results in differences in patient-reported outcomes and rates of repeat surgery over a 5-year follow-up.

**Methods:**

Follow-up 5 years after a randomized clinical trial, utilizing primary data collection linked to administrative health data, was carried out to create a longitudinal cohort. The primary outcome was participant-reported bothersome SUI symptoms, as defined by the Urogenital Distress Inventory (UDI-6) questionnaire. Secondary outcomes included participant-reported bothersome overactive bladder (OAB) scores, median scores of three validated urinary symptom questionnaires, and rates of subsequent surgery determined through patient report and administrative data.

**Results:**

Two hundred and sixty (81.8%) of the original study participants provided participant-reported data at 5 years. Administrative data linkage was completed for all of the original participants (*n* = 318). Demographic characteristics remained similar in the two groups at the 5-year follow-up mark. No differences existed in the primary outcome of reported bothersome SUI symptoms (30.8% Scissors vs 26.8% Babcock, *p* = 0.559), proportion of participants with bothersome OAB, the median scores of three validated bladder questionnaires, or in rates and cumulative incidence of recurrent MUS surgery or surgical revision of mesh-related complications.

**Conclusion:**

Both the Scissor and Babcock tensioning techniques provided comparable outcomes at 5 years post-MUS surgery. The information from this study allows surgeons to better decide which technique to adopt in their practice, providing confidence in longer-term cure and safety.

**Supplementary Information:**

The online version contains supplementary material available at 10.1007/s00192-024-05916-y.

## Introduction

Retropubic mid-urethral sling (MUS) surgical techniques vary among surgeons, with use of a Mayo Scissor as a spacer being most commonly used [1, 2]. There is limited research on optimal tensioning methods for achieving favorable patient outcomes [1, 3, 4]; existing studies assessing intra-operative sling tensioning primarily focus on outcomes at 1 year. A retrospective chart review comparing Kelly clamp versus Babcock clamp tensioning found similar rates of post-operative urinary retention and surgical revision, though without reporting on stress urinary incontinence resolution [4]. A subsequent randomized clinical trial (RCT), the "MUST trial," demonstrated that use of a Babcock clamp holding a fixed loop of the sling to determine tensioning resulted in comparable objective and subjective SUI cure rates with the more commonly known Mayo Scissor spacer tensioning, with lower rates of intervention for post-operative bladder outlet obstruction and overactive bladder (OAB) symptoms, but a potential increase in vaginal mesh erosion [3].

These findings suggest that the Babcock clamp technique applies less tension, yet it remains unclear if this translates to discrepancies in longevity between the techniques, given the known decline in MUS cure rates over time [5–7]. There is debate regarding degradation of polypropylene mesh after implantation [8–10], leaving the possibility that failed MUS over time could be partially due to loss of structural integrity. Additionally, given that age has been implicated in recurrent SUI [11], failure of MUS over time could be resultant of development of intrinsic sphincter deficiency or generalized age-related weakening of the urethral sphincter in the years after MUS insertion. For both possibilities, a “tighter” MUS may provide benefit for long-term SUI cure, despite higher rates of post-operative urinary retention. Post-operative uroflowmetry indicated higher rates of subclinical bladder obstruction with the "Scissor" technique through slower mean flow rates. The likelihood of subsequent surgical intervention among patients exhibiting subtle obstruction such as this remains unknown, given that > 50% of MUS revision surgeries occur more than 1 year post-insertion [5]. Moreover, sustained increases in post-MUS vaginal erosion rates have not been observed since the original MUST trial, which has prompted inquiries into the origin of the discrepancies in this outcome seen in the initial study.

We initiated a cohort study to conduct a 5-year follow-up on the original MUST trial participants. We hypothesized that the “Scissor” technique would have higher rates of self-reported SUI cure at the 5-year follow-up. Goals of the study were to ascertain whether differences between the two tensioning techniques developed with respect to SUI cure, if differences existed between tensioning groups with respect to repeat surgical intervention, and to explore possible reasons for the elevating vaginal erosion rate seen in the original trial.

## Materials and Methods

All 318 participants included in the original trial were eligible for cohort follow-up. Participant characteristics and details of the original surgical technique have been previously published [3]. Original participants were contacted approximately 2 months before their 5-year surgical anniversary to inform them of the follow-up study and to seek consent to repeat participation. Given that the 5-year follow-up window occurred during the COVID-19 pandemic, the cohort was developed with primary data collection being collected and managed using REDCap electronic data capture tools hosted at the Clinical Research Unit (CRU), Cumming School of Medicine, University of Calgary, Calgary, Alberta, Canada [12, 13]. For those participants who preferred to complete paper copies of the questionnaires, these were delivered and returned by postal mail with subsequent data entry into REDCap. This approach avoided in-person contact as there was a moratorium on in-person research visits within the relevant university and health care institutions. The original MUST RCT utilized an in-person examination to objectively measure the presence or absence of urine loss using a standardized cough-stress test physical examination [3]. Given the aforementioned COVID-19-related organizational directives to minimize unnecessary physical contact, we were unable to include this outcome in this study. However, results from previous MUS trials demonstrate high correlation between patient-reported and clinician-measured outcomes of SUI [3, 7, 14], supporting the decision that patient-reported outcomes in isolation would generate important data relevant to clinical decision making.

To ensure that all surgical interventions related to the original MUS insertion were included in the analysis, and to limit recall and attrition biases, administrative health data were linked to all original 318 trial participants. Administrative data sources included the Discharge Abstract Database (DAD), which captures all inpatient surgical procedures and hospital admissions, and the National Ambulatory Care Reporting System (NACRS), which captures all same-day surgeries and emergency room visits. These datasets adhere to International Classification of Diseases, Canadian Version-10, and the Canadian Classification of Health Intervention standards of coding [15, 16], and have previously been utilized to study longer term outcomes of MUS surgery in Canada [5, 17, 18].

Participants maintained classification by the randomization groups from the original study. Group demographics were compared by absolute standard difference (ASD) to test for a continued balance between randomization groups [19]. In keeping with the original trial, the primary outcome for the 5-year follow-up was bothersome versus nonbothersome SUI symptoms, defined by a score of ≥ 2 on Question 3 of the Urinary Distress Inventory (UDI-6) [3, 20]. Secondary outcomes included bothersome versus nonbothersome OAB symptoms, as defined by a score of ≥ 2 on either Question 1 or Question 2 of the UDI-6; summary scores of the UDI-6, Incontinence Impact Questionnaire (IIQ-7) [20], and International Consultation on Incontinence Questionnaire Female Lower Urinary Tract Symptoms Long Form (ICIQ-FLUTS LF) module questionnaires [21]; as well as the rate and timing of repeat surgery related to SUI symptoms and/or complications from the original mid-urethral sling. Mean difference (MD) and 95% confidence interval (CI) were calculated for proportional and numerical data; repeat surgeries were expressed as both incidence rate and cumulative incidence, as defined by the time between the original MUS surgery and the subsequent surgery [5].

Subsequent surgery for SUI and/or mesh complications was determined through patient report and administrative data. Validated Canadian Classification of Health Intervention (CCI) codes, which indicated a subsequent surgery for SUI treatment and/or to remove or revise implanted surgical devices or mesh, removal of a urethral foreign body, urethral dilation, retropubic or transvaginal urethrolysis, or repair of a urethrovaginal fistula were used to flag subsequent surgery in administrative data [5]. After identification of subsequent surgery by either participant report and/or occurrence of a validated code in the administrative data sources, the specific case was confirmed by chart review to determine the nature of the procedure confirmed. Descriptive statistics reported the frequencies of diagnostic International Statistical Classification of Diseases and Related Health Problems, 10th Revision, Canada (ICD-10-CA) codes and the CCI codes represented by these procedures.

A manual review of all mesh erosion cases recorded in the dataset was performed, in response to questions that have arisen since the original MUST paper as to explanation of mesh erosions in the original Babcock group that were not sustained after conclusion of the RCT. Given that Babcock was a novel technique for the study surgeons, we classified each Babcock case relative to the surgeon’s first Babcock case in the trial to explore for any temporal relationship in mesh erosion and introduction of the Babcock technique into a surgeon’s practice.

An a priori power calculation was performed for this study’s primary outcome, assuming a follow-up rate of 75% at 5 years (*n* = 238 over both arms) based on prior MUS follow-up studies [7], as well as an SUI cure rate of 69% at 5 years using Question 1 of the UDI-6 [7] and a non-inferiority limit of 15% [22], which has previously been determined as the threshold for surgeons to favor one surgical approach to the treatment of SUI over another [1, 14]. Calculations revealed that a sample of this size would have > 80% power with alpha = 0.05 to exclude a difference in participant-reported SUI cure.

Although all the original trial participants are biologically female, the original MUST trial methodology did not collect self-identified gender as part of demographic characteristics. Realizing this oversight, at the 5-year follow-up we did collect self-reported gender identity, as well as a measure of gender expression, in alignment with expert opinion that incorporation of the complexities of gender should be improved in health research [23, 24]. Given that within a gender identity, an individual may report differing levels of femininity and masculinity, measurement of gender expression is an analytic approach to better understanding how the nuances of gender are connected to experiences of health [25]. A secondary analysis of gender expression as a binary variable of gender polar with exclusively feminine traits (only feminine scores reported, masculine score of zero) or diversity in gender expression with mixed feminine and masculine traits (≥ 1 feminine score, ≥ 1 masculine score) was planned given that gender expression has previously been associated with patient perceptions in pelvic floor disorders [25]. Additionally, sensitivity analyses using a more conservative definition of bothersome SUI and OAB symptoms as defined by a score of ≥ 1 on corresponding questions of the UDI-6 were planned to ensure robust findings.

The original study protocol was registered at ClinicalTrials.gov (NCT02480231), and inclusion/exclusion criteria and technique details have been previously described [1, 3]. This study obtained ethics approval from the relevant university ethics board. The Strengthening the Reporting of Observational Studies in Epidemiology (STROBE) statement for reporting of observational studies was followed [26].

## Results

**Fig. 1:**
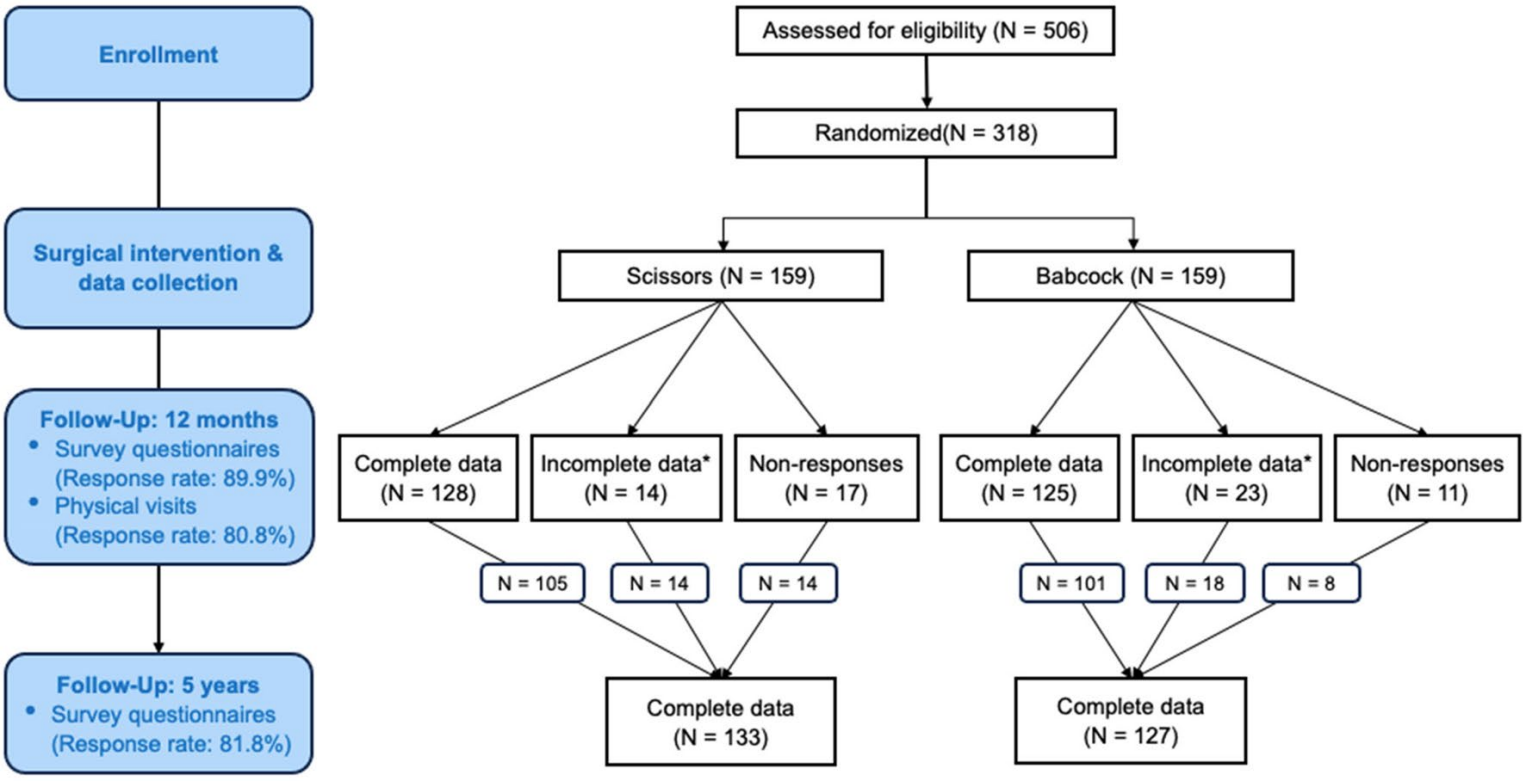
Consolidated Standards of Reporting Trials flow diagram. *Asterisk* indicates responses to at least one outcome

The follow-up cohort recruited 260 (81.8%) of the original participants to complete questionnaires at 5 years post-surgery. The most common reason for nonparticipation was the inability to make contact with the original participant. Of these participants, 22 were considered lost to follow-up at 12 months but opted to provide follow-up data at 5 years post-surgery (Fig. 1). There was no difference in the proportion of individuals completing self-reported questionnaires at 5 years between the original Babcock (*n* = 127) and the Scissor (*n* = 133) groups (*p* = 0.759). Administrative data linkage was available for all of the 318 original participants.

At 5 years post-surgery, the only demographic that statistically differed between groups was marital status, with a higher proportion of participants in the Scissor group now reporting being divorced, separated, or widowed (*p* = 0.049). As information on marital status was not collected for the original trial, it is unknown if this difference was present at allocation or whether it occurred as a change in relationship status over time. The overall balance in participant characteristics demonstrated that the randomization from 5 years prior was still valid to address confounding. At 5-year follow-up, all participants self-reported their gender as “woman” (Table 1).

**Table 1:** Patient characteristics at baseline and 5-year follow-up by two mid-urethral sling tensioning techniques (*N* = 260)

Characteristic	Scissor (*N* = 133)		Babcock (*N* = 127)		ASD
	n	%	n	%	
At 5-year follow-up					
Age (years), mean (SD)	57.7	(10.9)	57.1	(11.1)	0.06
Body mass index (kg/m^2^), mean (SD)	29.1	(7.1)	28.2	(5.8)	0.14
Race/ethnicity					
BIPOC	15	11.3	16	12.6	0.04
White	118	88.7	110	87.3	0.04
Marital status					
Single	8	6.0	8	6.3	0.01
Married/common-law	86	64.7	98	77.2	0.28
Divorced/separated/widowed	39	29.3	21	16.5	0.31
Gender identity					
Woman	133	100.0	127	100.0	0.00
Gender expression					
Femininity					
Mean (SD)	5.2	(1.1)	5.3	(1.1)	0.06
Median (IQR)	6	(5, 6)	6	(5, 6)	–
Masculinity					
Mean (SD)	0.5	(1.1)	0.5	(1.0)	0.01
Median (IQR)	0	(0, 1)	0	(0, 1)	–
Smoking status					
Never	71	53.4	66	52.0	0.03
Past	48	36.1	51	40.2	0.08
Current	14	10.5	10	7.9	0.09
Menopause status					
Post-menopause	95	71.4	84	66.1	0.11
Parity					
0	3	2.3	7	5.5	0.17
1	19	14.7	17	14.3	0.01
2	63	48.8	51	42.9	0.12
3 +	47	36.4	51	42.9	0.13
Number of vaginal births					
0	10	7.5	15	11.8	0.15
1	24	18.0	19	15.0	0.08
2 +	99	74.4	93	73.2	0.03
Hormone therapy use					
Never	60	63.2	54	64.3	0.02
Past	17	17.9	13	15.5	0.06
Current	18	18.9	17	20.2	0.03

1 missing for race/ethnicity, 1 missing for gender identity, 3 missing for gender expression of femininity, 4 missing for gender expression of masculinity, 12 missing for parity, 81 missing for hormone therapy use, 2 missing for concomitant prolapse surgery, 1 missing for IIQ-7 scores, 3 missing for ICIQ-FLUTS LF scores. Statistics were based on available data

*BIPOC* Black, Indigenous, and people of color, *SD* standard deviation, *IQR* interquartile range, *UDI-6* Urinary Distress Index, *IIQ-7* Incontinence Impact Questionnaire, *ICIQ-FLUTS LF* International Consultation on Incontinence Questionnaire Female Lower Urinary Tract Symptoms Long Form, *ASD* absolute standardized difference

No statistically significant differences existed in the primary and secondary outcomes, nor in the sensitivity analysis using a more conservative definition of OAB and SUI symptoms (Table 2). Analysis stratified by gender expressions indicated a lower proportion of bothersome OAB symptoms (*p* = 0.022) and lower median score of ICIQ-FLUTS LF (*p* = 0.039) in the Babcock group among gender polar women (Table 3).

**Table 2:** Primary outcomes at 5-year follow-up (*N* = 260)

Outcome	Scissor (*N* = 133)	Babcock (*N* = 127)	Mean difference^a^ (95% confidence interval)
Symptoms			
SUI, *n* (%)			
Nonbothersome symptoms	92 (69.2)	93 (73.2)	4.06 (−6.94, 15.05)
Bothersome symptoms (2 or greater)	41 (30.8)	34 (26.8)	−4.06 (−15.05, 6.94)
OAB, *n* (%)			
Nonbothersome symptoms	63 (47.4)	74 (58.3)	10.90 (−1.17, 22.96)
Bothersome symptoms	70 (52.6)	53 (41.7)	−10.90 (−22.96, 1.17)
Questionnaire scores			
UDI-6			
Mean (SD)	33.0 (23.2)	30.6 (23.6)	−2.40 (−8.09, 3.29)
Median (IQR)	27.8 (16.7, 50.0)	27.8 (11.1, 50.0)	–
IIQ-7			
Mean (SD)	16.8 (25.7)	14.9 (23.4)	−1.92 (−7.89, 4.04)
Median (IQR)	4.8 (0.0, 23.8)	0.0 (0.0, 21.4)	–
ICIQ-FLUTS LF			
Mean (SD)	17.0 (10.2)	15.3 (8.9)	−1.73 (−4.06, 0.60)
Median (IQR)	15.0 (9.0, 23.0)	14.0 (8.0, 21.0)	–

SUI was defined by UDI-6 Q3 (“How much are you bothered by urine leakage related to coughing, sneezing, or laughing?”) Bothersome SUI symptoms were defined as a score of 2 or greater

OAB was defined by UDI-6 Q1 (“How much are you bothered by frequent urination?”) and Q2 (“How much are you bothered by urine leakage associates with a feeling of urgency…?”). Bothersome OAB symptoms were defined based on a score of 2 or greater for either question

*SUI* stress urinary incontinence, *OAB* overactive bladder, *IQR* interquartile range, *UDI-6* Urinary Distress Index, *IIQ-7* Incontinence Impact Questionnaire, *ICIQ-FLUTS LF* International Consultation on Incontinence Questionnaire Female Lower Urinary Tract Symptoms Long Form

^a^Difference in proportions for categorical outcomes and difference in mean values for continuous outcomes

**Table 3:** Primary outcomes at 5-year follow-up stratified by gender expressions (*N* = 256^a^)

Outcome	Gender polar (*N* = 185)			Gender diverse (*N* = 71)		
	Scissor (*N* = 94)	Babcock (*N* = 91)	MD (95% CI)	Scissor (*N* = 36)	Babcock (*N* = 35)	MD (95% CI)
Symptoms						
SUI, *n* (%)						
Nonbothersome symptoms	64 (68.1)	70 (76.9)	8.84 (−3.96, 21.63)	26 (72.2)	23 (65.7)	−6.51 (−27.99, 14.97)
Bothersome symptoms	30 (31.9)	21 (23.1)	−8.84 (−21.63, 3.96)	10 (27.8)	12 (34.3)	6.51 (−14.97, 27.99)
OAB, *n* (%)						
Nonbothersome symptoms	40 (42.6)	55 (60.4)	17.89 (3.71, 32.06)	21 (58.3)	19 (54.3)	−4.05 (−27.11, 19.01)
Bothersome symptoms	54 (57.4)	36 (39.6)	−17.89 (−32.06, −3.71)	15 (41.7)	16 (45.7)	4.05 (−19.01, 27.11)
Questionnaire scores						
UDI-6						
Mean (SD)	34.2 (23.3)	28.9 (22.5)	−5.33 (−11.93, 1.28)	30.6 (22.6)	33.7 (25.2)	3.13 (−8.02, 14.28)
Median (IQR)	33.3 (16.7, 50.0)	22.2 (11.1, 50.0)	–	27.8 (15.3, 45.8)	33.3 (11.1, 47.2)	–
IIQ-7						
Mean (SD)	16.6 (25.4)	12.2 (20.6)	−4.40 (−11.05, 2.25)	17.6 (27.1)	20.7 (28.1)	3.09 (−9.76, 15.94)
Median (IQR)	4.8 (0.0, 22.6)	0.0 (0.0, 14.3)	–	4.8 (0.0, 23.8)	9.5 (0.0, 33.3)	–
ICIQ-FLUTS LF						
Mean (SD)	17.7 (10.5)	14.4 (8.5)	−3.33 (−6.07, −0.60)	15.5 (9.5)	17.4 (9.8)	1.96 (−2.53, 6.46)
Median (IQR)	16.0 (10.0, 23.8)	13.0 (8.0, 18.5)	–	13.5 (8.0, 21.2)	18.0 (10.0, 25.0)	–

Gender polar was defined as a positive score of self-reported femininity and a zero or missing score of self-reported masculinity; gender diverse was defined as positive scores of both femininity and masculinity

*SUI* stress urinary incontinence, *OAB* overactive bladder, *SD* standard deviation, *IQR* interquartile range, *UDI-6* Urinary Distress Index, *IIQ-7* Incontinence Impact Questionnaire, *ICIQ-FLUTS LF* International Consultation on Incontinence Questionnaire Female Lower Urinary Tract Symptoms Long Form, *MD* mean difference, *CI* confidence interval

^a^4 participants with missing gender polarity were excluded

There were 29 administrative data records indicating subsequent SUI- and/or MUS-related surgical intervention after index MUS and 11 reported by participants as related to their original MUS and/or recurrent SUI. This represented 29 individuals, of whom 8 underwent multiple SUI- and/or MUS-related surgical procedures in the 5-year follow-up window. Analysis of repeat surgeries demonstrated similar cumulative incidence of repeat surgery at 5-year follow-up, with the majority of cases occurring in the 1st year after the original procedure (Table 4). Manual review of the repeat surgical intervention revealed that mesh erosions occurred for 4 of the original 7 study surgeons, notably excluding those surgeons with the highest volume of cases in the trial. A pattern was demonstrated with all cases resulting in vaginal mesh erosion occurring within the 1st year of the attending surgeon’s first Babcock case. Notably, 71.4% of the “mesh erosion” cases took place within the first 6 months of the attending surgeon’s index Babcock-tensioning case.

**Table 4:** Incidence rates of subsequent surgeries up to 5 years post-MUS tensioning by Scissor and Babcock (*N* = 318)

Interval of time post-index MUS surgery	Scissors (*N* = 159)				Babcock (*N* = 159)			
	Number of patients		Incidence rate^a^, per 100 person years (95% CI)	Cumulative incidence^b^, % (95% CI)	Number of patients		Incidence rate, per 100 person years (95% CI)	Cumulative incidence^b^, % (95% CI)
	At start of interval	With recurrent MUS			At start of interval	With recurrent MUS		
Recurrent SUI surgeries								
30 days or less	159	0	0.00 (0.00, 0.00)	0.00 (0.00, 0.00)	159	0	0.00 (0.00, 0.00)	0.00 (0.00, 0.00)
30 days to 1 year	159	2	1.38 (0.00, 3.28)	1.26 (0.00, 2.98)	159	2	1.38 (0.00, 3.28)	1.26 (0.00, 2.98)
1–2 years	157	2	1.28 (0.00, 3.05)	2.52 (0.05, 4.92)	157	1	0.64 (0.00, 1.89)	1.89 (0.00, 3.98)
2–3 years	155	2	1.30 (0.00, 3.09)	3.77 (0.77, 6.69)	156	2	1.29 (0.00, 3.07)	3.14 (0.39, 5.82)
3–4 years	153	0	0.00 (0.00, 0.00)	3.77 (0.77, 6.69)	154	0	0.00 (0.00, 0.00)	3.14 (0.39, 5.82)
4–5 years	153	0	0.00 (0.00, 0.00)	3.77 (0.77, 6.69)	154	0	0.00 (0.00, 0.00)	3.14 (0.39, 5.82)
Mesh complications								
30 days or less	159	8	62.84 (36.29, 89.38)	5.03 (1.57, 8.37)	159	2	15.41 (0.00, 35.06)	1.26 (0.00, 2.98)
30 days to 1 year	151	1	0.72 (0.00, 2.14)	5.66 (2.00, 9.18)	156	4	2.81 (0.10, 5.53)	3.77 (0.77, 6.69)
1–2 years	150	0	0.00 (0.00, 0.00)	5.66 (2.00, 9.18)	153	3	1.98 (0.00, 4.20)	5.66 (2.00, 9.18)
2–3 years	150	0	0.00 (0.00, 0.00)	5.66 (2.00, 9.18)	150	0	0.00 (0.00, 0.00)	5.66 (2.00, 9.18)
3–4 years	150	1	0.67 (0.00, 1.98)	6.29 (2.44, 9.99)	150	1	0.67 (0.00, 1.98)	6.29 (2.44, 9.99)
4–5 years	149	0	0.00 (0.00, 0.00)	6.29 (2.44, 9.99)	149	1	0.67 (0.00, 1.99)	6.92 (2.89, 10.78)

Three participants contributed to both recurrent SUI surgeries and mesh complications

^a^Incidence rate during the interval: number of new cases/total person-time at risk based on average sample size during the follow-up period

^b^Cumulative incidence at the end of the interval: number of new cases/total number of patients at risk at the beginning of the follow-up period

## Discussion

This follow-up of the MUST trial compared prevalence of bothersome SUI symptoms at 5 years after retropubic MUS surgery tensioned by the Mayo Scissor versus the Babcock clamp technique. In addition to self-reported SUI symptoms, median scores of three validated bladder questionnaires and rates of subsequent surgery related to the participant’s original MUS procedures and/or on-going SUI symptoms were additionally compared. No differences were found between the two groups; rates of self-reported SUI cure at 5 years remained high.

Strengths of this study include longer-term follow-up of an RCT. Exploration of participant demographics at 5 years demonstrated minimal differences between the two groups, suggesting that analyzing within the original allocation was still valid and that the randomized approach would continue to mitigate confounding. Although follow-up at 12 months is the most common follow-up period for MUS RCTs [27], we designed this longer-term study in response to the data paucity and resulting calls for longer follow-up studies as “essential” to understanding the benefits and risk of MUS [27]. Additional strengths include the ability to engage participants previously lost to follow-up at 12 months, and the universal linkage of administrative data to all original participants, reducing attrition and recall bias. Limitations of the study include the lack of in-person follow-up. Although previous work at our institution suggests high correlation between subjective participant-reported and objective cure defined by standardized cough stress testing [3, 7, 14], the lack of an in-person study visit precluded the ability to obtain a physical examination and collect the uroflowmetry parameters, which were part of the original trial [3]. Owing to the recruitment profile of the original RCT, this follow-up may have limited generalizability to very young patients, those with extreme body habitus, marginalized populations, and patients with more complex incontinence or prior SUI surgeries. The secondary analysis by gender expression improves generalizability through recognition of the fact that expressions of femininity and masculinity varies among cisgender women; the finding of a difference in OAB scores in the Babcock arm requires further research to understand how one’s self-expression intersects with lower urinary tract symptoms.

This study also builds upon the existing literature on retropubic MUS tensioning [3, 4], demonstrating that cure of SUI symptoms at 5 years is not associated with the specific tensioning technique. Differences in proportions of individuals reporting OAB in the original 12-month study subsequently attenuated and were no longer statistically significant in the 5-year data. Of note, > 10% of participants reported bothersome OAB at the 12-month follow-up, yet at 47.3%, the proportion was noticeably higher 5 years later. This raises the possibility that health changes in the years following MUS insertion, such as those related to aging [28, 29], may have a greater impact on OAB rates than the MUS tensioning technique.

This study offers important insights into surgical decision making. Given that urinary retention may require additional interventions such as catheterization or sling loosening, [30], which results in additional burden on the patient, the favorable findings of reduced post-operative bladder outlet obstruction with the Babcock clamp in the original MUST trial are now bolstered by this study’s reassuring data on 5-year efficacy. As both tensioning techniques were found to have similar 5-year outcomes, continence rates, and patient satisfaction, surgeons can balance this information with their comfort managing post-operative urinary retention to make informed choices regarding sling tensioning methods and optimize individual patient outcomes and satisfaction following MUS procedures.

Additionally, this study suggests a mechanism to explain the signal regarding vaginal mesh erosion reported in the original MUST trial. Mesh erosion cases appeared to have occurred early in a surgeon’s exposure to the Babcock technique. The team’s interpretation is that mesh erosion rates declined after surgeons began “tapping” the MUS mesh into the sub-urethral space upon releasing the Babcock clamp, prior to incision closure. This purposeful step was adopted while the MUST trial was underway, as it effectively flattens the mesh loop and moves the mesh out of direct contact with the vaginal epithelium. Since conclusion of the MUST trial, the Babcock technique has become the prevalent tensioning mechanism at the study’s main institution, without evidence of elevated mesh erosion rates. The study team hypothesizes that the cause of erosions might have been the proximity of the folded mesh upon immediate release of the clamp to the healing incision and/or the edge of the MUS being non-intentionally caught in the suture closure. Given that the reproducible aspects of the Babcock technique are advocated to be of most benefit to surgeons earlier in their career, or those who perform a low volume of MUS, a video of the technique has been developed that includes this “tapping” step (Video).

In conclusion, both the Scissor and Babcock techniques for tensioning retropubic MUS yield comparable and favorable rates of SUI cure at 5 years. Furthermore, there is no sustained discrepancy in symptoms of OAB, disease-specific quality-of-life scores, or the need for subsequent surgeries between the two techniques. Given that the Babcock technique demonstrates lower rates of immediate post-operative urinary retention, there are potential early post-operative benefits to its use and surgeons can confidently consider incorporating the Babcock technique into their MUS practice as an additional intra-operative tensioning option.

## Supplementary Information

Below are the links to the electronic supplementary material.

Supplementary file1 (DOCX 36 KB)

Supplementary file2 (PDF 110 KB)

## Data Availability

*Will individual participant data be available (including data dictionaries)?* Yes. *What data in particular will be shared?* Individual participant data that underlie the results reported in this article, after de-identification. *What other documents will be available?* Study protocol, statistical analysis plan. *When will data be available (start and end dates)?* Beginning 12 months and ending 36 months following article publication. *By what access criteria will data be shared (including with whom, for what types of analyses, and by what mechanism)?* Following approval by a relevant institutional review board of a methodologically sound proposal and signing of an inter-organization data-sharing agreement, data will be available directly from the lead author to investigators at academic institutions.
