# Supplementary material for: Babcock versus Scissor Tensioning for Retropubic Mid-Urethral Slings: Comparing Two Intra-Operative Techniques Through 5 Years of Follow-Up
Source: Int Urogynecol J. 2024 Oct 1;36(2):279–87. doi: 10.1007/s00192-024-05916-y (PMC11895215; doi:10.1007/s00192-024-05916-y)
Supplement: Supplementary file 2 — Supplementary file2 (PDF 110 KB) [file 192_2024_5916_MOESM2_ESM.pdf]

Supplementary:

eTable 1. Frequencies of Canadian Classification of Health Intervention (CCI) codes representing subsequent surgeries up to 5 years post-MUS tensioning by Scissor and Babcock (N = 318)

CCI code / Chart Review	Description	Scissor (N = 159)		Babcock (N = 159)	
		n	%	n	%
**Surgery for recurrent urinary incontinence**					
1.PL.74.AL-XX-N	Fixation, bladder neck combined per orifice (vaginal) and percutaneous approach using synthetic material (e.g., TVT technique)	1	0.6	2	1.3
1.PL.74.CR-XX-N	Fixation, bladder neck, per orifice (vaginal) approach with incision using synthetic tissue (e.g., TVT, Monarc, SPARC)	1	0.6	1	0.6
1.PL.74.AF-XX-N	Fixation, bladder neck combined per orifice (vaginal) and open (abdominal) approach using synthetic material	--	--	--	--
1.PL.74.LA-XX-N	Fixation, bladder neck open, perineal approach using synthetic material (e.g., laparotomy, pubovaginal sling)	--	--	--	--
1.PL.74.DA-XX-N	Fixation, bladder neck endoscopic (laparoscopic) approach using synthetic material (e.g., laparoscopic procedure at time of TVT, laparoscopic mesh sling)	--	--	--	--
Charts review	--	4	2.5	2	1.3
Total	--	6	3.8	5	3.1
**Surgery for MUS complications**					
1.PL.54.CA-XX-N	Management of internal device, bladder neck of synthetic urethral sling (TVT) using per orifice vaginal approach	5	3.1	8	5.0
1.PL.54.LA-XX-N	Management of internal device, bladder neck of synthetic material (urethral sling) (TVT) using open laparotomy approach	1	0.6	0	0.0
1.PL.54.LB-PZ	Management of internal device, bladder neck, of artificial sphincter using open approach	--	--	--	--
1.PL55.CA-XX-N	Removal of device, bladder neck of synthetic urethral sling (TVT) using vaginal approach	1	0.6	0	0.0
1.PL.55.LA-XX-N	Removal of device, bladder neck of synthetic urethral sling (TVT) using open laparotomy approach	--	--	--	--
1.PL.55.LB-PZ	Removal of device, bladder neck, of artificial sphincter using open approach	--	--	--	--
1.PQ.56.*	Removal of foreign body, any approach	--	--	--	--
1.PQ.57.*	Extraction of material from urethra, any approach	--	--	--	--
1.PQ.59.*	Destruction urethra, any approach	--	--	--	--
1.PQ.72.*	Release urethra, by any approach (e.g., urethrolysis)	--	--	--	--
1.PQ.86.*	Closure of fistula, urethra, by any approach	--	--	--	--
Charts review	--	3	1.9	3	1.9
Total	--	10	6.3	11	6.9

Three participants contributed to both recurrent SUI surgeries and mesh complications. Some participants were reclassified by chart review.

eTable 2. Sensitivity analysis of more conservative definition of bothersome SUI and OAB outcome

Outcome	Scissor (N = 133)	Babcock (N = 127)	Mean Difference* (95% Confidence Interval)
Symptoms			
SUI, n (%)			
Non-bothersome symptoms (0)	65 (48.9)	59 (46.5)	-2.42 (-14.56, 9.73)
Bothersome symptoms (1 or greater)	68 (51.1)	68 (53.5)	2.42 (-9.73, 14.56)
OAB, n (%)			
Non-bothersome symptoms (0)	22 (16.5)	25 (19.7)	3.14 (-6.22, 12.51)
Bothersome symptoms (1 or greater)	111 (83.5)	102 (80.3)	-3.14 (-12.51, 6.22)

SUI was defined by UDI6-Q3 ("How much are you bothered by urine leakage related to coughing, sneezing, or laughing?") For this sensitivity analysis, bothersome SUI symptom was defined as a score of 1 or greater.

OAB was defined by UDI6-Q1 ("How much are you bothered by frequent urination?") & Q2 ("How much are you bothered by urine leakage associates with a feeling of urgency…?"). For this sensitivity analysis, bothersome OAB symptom was defined based on a score of 1 or greater for either question.
